# Irregular Findings on Teatcups in Milking Parlours in Sheep and Goat Farms and Potential Predictors

**DOI:** 10.3390/ani13193004

**Published:** 2023-09-23

**Authors:** Charalambia K. Michael, Daphne T. Lianou, Vasia S. Mavrogianni, Efthymia Petinaki, George C. Fthenakis

**Affiliations:** 1 Veterinary Faculty, University of Thessaly, 43100 Karditsa, Greece; 2 University Hospital of Larissa, 41110 Larissa, Greece

**Keywords:** cleaning, goat, mastitis, milk, milking parlour, sheep, *Staphylococcus*, teatcup

## Abstract

**Simple Summary:**

This study investigated the presence of irregular findings on teatcups in milking parlours in sheep and goat farms in Greece. It also referred to the description of these findings and the elucidation of factors possibly associated with their presence and was performed in 255 sheep and 66 goat farms in Greece. In almost half of the farms, teatcups with irregular findings were found in the respective milking parlours. These findings included dirt, milk residues and cracks or tears on the teatcups. Bacteria were more frequently isolated from such teatcups in comparison to undamaged teatcups. The following variables related to the management in the farms emerged as significantly associated with the presence of irregular findings on the teatcups: the daily number of milking sessions, month into the lactation period at sampling and number of available milking units per animal position in the parlour.

**Abstract:**

The objectives of the present study were as follows: (a) to describe the prevalence of irregular findings on teatcups in milking parlours with dairy sheep and goats after the post-milking cleaning procedures had been completed, (b) to associate staphylococcal isolation from teatcups with the presence with irregular findings and (c) to identify predictors of the presence of irregular findings on teatcups. The teatcups in the milking parlour of 255 sheep and 66 goat farms were macroscopically evaluated for the presence of irregular findings immediately after the completion of cleaning of the parlour. In total, 1115 and 303 teatcups, respectively, were assessed. A detailed interview with the farmer served to record the characteristics of the milking parlour and obtain information about husbandry and health management variables in the farm. Teatcups with macroscopically evident irregular findings were observed in 150 milking parlours (46.7%). Overall, 593 teatcups (41.8%) were found with macroscopically evident irregular findings. Dirt, milk residues and cracks or tears were recorded in the teatcups of 90.0%, 36.0% and 12.7% of parlours with irregular findings. Staphylococci were more frequently isolated from teatcups with irregular findings than from undamaged ones: from 37.4% (222/593) versus 12.8% (106/825). They were more frequently isolated from teatcups with milk residues (39.4%) and teatcups with dirt (39.0%). Via multivariable analysis, the following three variables emerged as significant predictors of presence of teatcups with irregular findings: the daily number of milking sessions, month into the lactation period at sampling and number of available milking units per animal position. The study provides, for the first time internationally, an appraisal of the frequency of problematic teatcups in the milking parlours of small ruminant farms. The analysis of predictors has provided a focus for specific management aspects, where interventions need to be performed, in order to improve the situation in farms with problems. The consequent increased staphylococcal burden on teatcups with irregular findings points to the increased risk of intramammary infections in such cases.

## 1. Introduction

The cleaning of the milking system, after the completion of the milking procedure, is essential for the correct maintenance of the system and securing hygienic procedures during the milking of animals. The cleaning of the system includes the use of hot water and chemicals (detergents), with the aim being to remove organic deposits on the teatcups and the liners of the milking system; these methods can be coupled with the scrubbing of the system’s parts and units, which has a mechanical effect in terms of removing residues [1].

The importance of effective post-operation cleaning of the milking system was previously recognised in the 1960s [2]. However, a recent (August 2023) topical search in Web of Science using the terms [‘milking parlour’ AND ‘clean *’] (the asterisk served as a truncation symbol) returned 83 items, though a detailed evaluation of these records, at individual basis, revealed that only 56 publications were relevant.

There is a consensus among these studies that the omission or incorrect application of the cleaning procedures in the milking system in dairy farms is associated with increased numbers of bacteria in milk produced in such farms. The micro-organisms found to be in abundance in the milk produced in farms not applying appropriate cleaning procedures were staphylococci [3], streptococci [4], coliforms [5], *Bacillus* spp. spores [6] and yeasts [7]. This issue can possibly be explained by a build-up of milk residues in the system, which facilitate bacterial growth, thus contributing to increased microbial populations in the bulk-tank milk [1].

Nevertheless, the majority of the above studies (52 of 56) refer to dairy cattle farms, which indicates a clear paucity of relevant studies in sheep flocks and goat herds. In some parts of the world, however, milk production by small ruminants is particularly important; for example, in Greece, the annual milk production from these animals is higher than that from cattle: in 2022, annual milk production from small ruminants in Greece was 876,000 tons [8], whilst that from cattle amounted to 643,000 tons [9], a situation that makes Greece unique among European countries [10].

The objectives of the present study were (a) to describe the prevalence of irregular findings on teatcups in milking parlours with dairy sheep and goats after the post-milking cleaning procedures had been completed, (b) to associate staphylococcal isolation derived from teatcups with the presence with irregular findings and (c) to identify predictors of the presence of irregular findings on teatcups.

## 2. Materials and Methods

### 2.1. Sheep and Goat Farms

**Figure 1:**
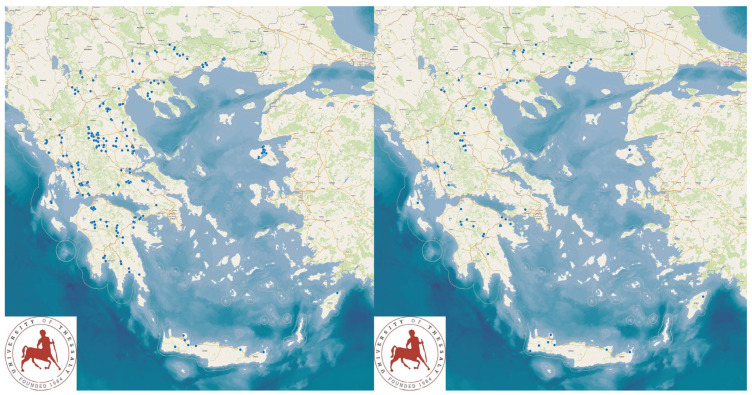
Location of the small ruminant farms (right map: sheep flocks; left map: goat herds) around Greece, which were visited for the evaluation of teatcups in milking parlours and for the collection of samples and information.

This work was performed as a cross-sectional study of 325 sheep and 119 goat farms throughout Greece, of which 255 and 66, respectively, had milking parlours. The farms were located throughout the country (Figure 1). All of the farms were visited for the macroscopic evaluation of teatcups in milking parlours, as well as for the collection of information and samples. The farms were selected based on the willingness of farmers to accept a visit by University personnel for an interview and sample collection. The main investigators (authors C.K.M. and G.C.F.) visited all of the participating farms.

A detailed interview with the farmer served to record the characteristics of the milking parlour and obtain information about husbandry-related variables and variables related to health management applied in the farm [11].

### 2.2. Evaluation of Teatcups

In each farm, the teatcups in the milking parlour were macroscopically evaluated through careful observation to describe any irregular findings. The below protocol was followed. In milking parlours with one milking unit (*n* = 4), both teatcups were evaluated; in parlours with two to 12 milking units (*n* = 189), three teatcups were evaluated; in parlours with 13 to 24 units (*n* = 109), six teatcups were evaluated; and, finally, in parlours with 25 to 36 (*n* = 13) or 37 to 48 units (*n* = 6), 9 and 12 teatcups, respectively, were evaluated.

The specific teatcups that were observed and evaluated in each parlour had been predetermined using an electronic random number generator. Therefore, in total, 1115 teatcups in sheep farms and 303 teatcups in goat herds were observed and evaluated.

The observations were performed after a milking session had finished and the parlour had been cleaned, with relevant procedures performed using the normal farm routine. In order to maintain uniformity, the observations were always made by the same person (C.K.M.). Any macroscopically evident irregular findings on the teatcups were recorded.

### 2.3. Collection of Samples and Laboratory Examinations

After the completion of the macroscopic assessment, two separate swabs were obtained from the same teatcups: one from the upper part of each teatcup (to a depth of approximately 1 to 1.5 cm) and one from the lower part of the teatcup (to a depth of approximately 10 to 12 cm). Sampling was carried out in a circular manner, and the entire inner wall of the teatcup was swabbed. Duplicate swab samples were obtained; hence, in total, four swabs were used for sampling on each teatcup. These swabs were then immediately immersed in medium for transportation (Liquid Based Microbiology—LBM; BioMerieux, Marcy-l’-Étoile, France).

The samples were packed at 0.0 to 4.0 °C. Transportation to the laboratory was carried out by the investigators. The swabs were processed for bacteriological examination.

In brief, the microbiological examinations started within 24 h of obtaining the samples. Each of the four swabs collected from the sampling from each teatcup (upper and lower part) was streaked in duplicate media, specifically on sheep blood (5%) agar and selective medium for *Staphylococcus* (mannitol agar). All of the plates were aerobically incubated at 37 °C for up to 72 h. The definitive identification (i.e., at species level) of the staphylococcal isolates obtained was made using matrix-assisted laser desorption/ionisation time-of-flight mass spectrometry (VITEK MS; BioMerieux, Marcy-l’-Étoile, France).

### 2.4. Data Management and Analysis

#### 2.4.1. Data Management

The irregular findings that were observed on teatcups during the macroscopic evaluation were grouped into three general types: (i) presence of dirt on the teatcups, (ii) presence of milk residues on the teatcups and (iii) presence of cracks or tears on the teatcups.

It was necessary to isolate and identify ≥ 3 colonies of staphylococci on the same agar plate as those streaked with each swab in order to confirm the presence of an organism. The upper part and the lower part of each teatcup were separately assessed. If staphylococci were recovered from either of these two parts of the teatcup, the teatcup was considered to be ‘contaminated’. For staphylococcal isolates that were similarly identified from samples obtained from the teatcups in a milking parlour on the same farm, they were deemed to be the same organism. Therefore, in such cases, they were only taken into account once in all relevant calculations.

#### 2.4.2. Statistical Analysis

Data were entered into Microsoft Excel and analysed using SPSS v. 21 (IBM Analytics, Armonk, NY, USA). Basic descriptive analysis was performed. Exact binomial confidence intervals (CIs) were obtained.

Comparisons between frequencies were performed using Pearson’s chi-square test or Fisher’s exact test as appropriate. Comparisons between continuous data were performed using the Mann–Whitney test or via analysis of variance (one-way or Kruskal–Wallis test), as appropriate.

In total, 33 variables related to (a) sampling conditions, (b) the general management in the flock / herd, (c) the milking parlour in the flock / herd and (d) the socio-demographic particulars of farmers were evaluated for the identification of predictors (Appendix A). Categories were created for these variables as appropriate, in accordance with the information provided by farmers during the interview.

The outcomes of the ‘presence of teatcups with irregular findings’, ‘presence of teatcups with dirt’, ‘presence of teatcups with milk residues’ and ‘presence of teatcups with cracks or tears’ were considered. Exact binomial CIs were obtained. The importance of predictors was assessed using cross-tabulation with Pearson’s chi-squared test and via simple logistic regression. Then, multivariable models were created. In these models, all variables that achieved *p* < 0.20 in the univariable analysis were offered to the model. Variables were removed from the initial model via backward elimination. The *p* value of the removal of a variable was assessed via the likelihood ratio test, and for those with a *p* value of > 0.20, the variable with the largest probability was removed. This process was repeated until no variable could be removed with a *p* value of > 0.20. The variables required for the various multivariable models are shown in Table S1.

In all analyses, statistical significance was defined at *p* < 0.05.

## 3. Results

### 3.1. Macroscopic Irregular Findings in Teatcups

Teatcups with macroscopically evident irregular findings were observed in 150 milking parlours (46.7%, 95% CIs: 41.3–51.2%). There was no difference between sheep and goat farms in terms of the proportion of milking parlours in which such teatcups were found: 46.7% (119/255) and 46.9% (31/66), respectively (*p* = 0.96).

Overall, 593 teatcups (41.8%, 95% CIs: 39.3–44.4%) were found with macroscopically evident irregular findings. The median value of the proportion of teatcups with irregular findings among those evaluated in a milking parlour was 0% (interquartile range: 100.0%). Once again, there was no difference between sheep and goat farms in the proportion of such teatcups in a milking parlour: 40.9% (456/1115) and 45.2% (137/303), respectively (*p* = 0.17).

The most frequently observed irregular finding was the presence of dirt on the teatcup: overall, it was found in 90.0% of milking parlours in which teatcups with irregular findings were found. Details are shown in Table 1.

### 3.2. Associations with Isolation of Staphylococci from Teatcups

Overall, staphylococci were isolated from 328 teatcups (23.1%, 95% CIs: 21.0%-25.4%). They were significantly more frequently isolated from teatcups with irregular findings than from undamaged (i.e., without irregular findings) ones: from 37.4% (222/593) versus 12.8% (106/825) of the respective teatcups (*p* < 0.0001). Staphylococci were more frequently isolated from teatcups with milk residues (39.4%) and teatcups with dirt (39.0%) than from teatcups with cracks or tears (23.1%) (*p* = 0.020).

There was no association between the type of irregular findings on the teatcups and the frequency of the isolation of the various staphylococcal species (*p* > 0.17). Details are shown in Table 2.

### 3.3. Variables Associated with Presence of Irregular Findings in Teatcups

#### 3.3.1. Teatcups with Macroscopic Irregular Findings

The detailed results of the univariable analysis of the presence of teatcups with macroscopic irregular findings are shown in Table S2. The statistical significance values of all of the variables assessed via the univariable analysis of the presence of irregular findings on teatcups are shown in Appendix B.

In the multivariable analysis, the following three variables emerged with a significance: (a) the daily number of milking sessions (*p* = 0.033), (b) month into the lactation period at sampling (*p* = 0.046) and (c) number of available milking units per animal position (*p* = 0.047) (Table 3).

Moreover, there was a clear correlation between the proportion of teatcups with irregular findings in a milking parlour and the above three variables that emerged as significant with the presence of such teatcups: *r_sp_* = 0.128 (*p* = 0.022), *r_sp_* = 0.138 (*p* = 0.013) and *r_sp_* = −0.175 (*p* = 0.002), respectively.

#### 3.3.2. Teatcups with Dirt

**Figure 2:**
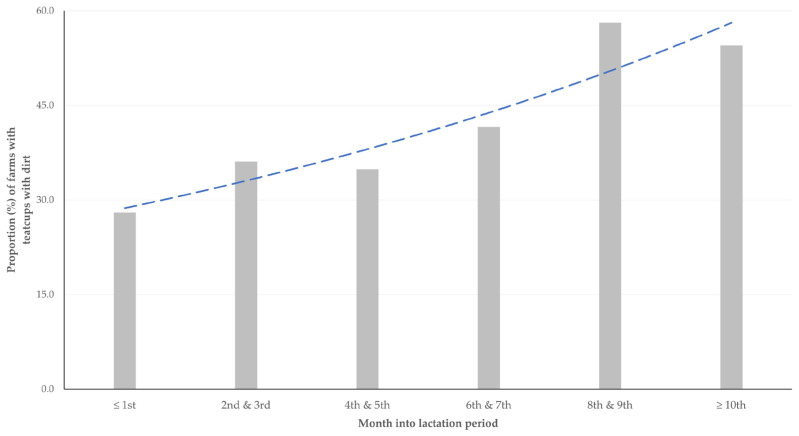
Proportion of sheep and goat farms in milking parlours in which teatcups with dirt were observed, in accordance with the month into the lactation period at each farm at the time of the visit (dashed line indicates tendency line).

The detailed results of the univariable analysis of the presence of teatcups with dirt are shown in Table S3. In the multivariable analysis, the following variable emerged with significance: the month into the lactation period at sampling (per unit increase (odds risk (±standard error): 1.029 ± 1.001) (*p* = 0.003) (Figure 2).

#### 3.3.3. Teatcups with Milk Residues

**Figure 3:**
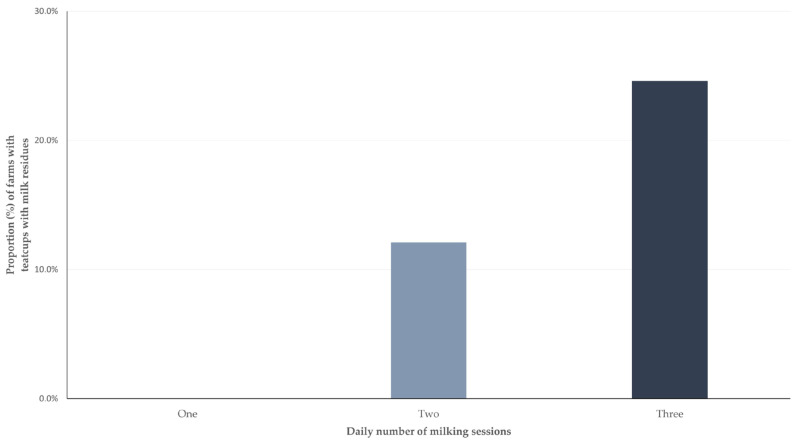
Proportion of sheep and goat farms in milking parlours in which teatcups with milk residues were observed, in accordance with the daily number of milking sessions performed in the farm.

The detailed results of the univariable analysis for the presence of teatcups with milk residues are shown in Table S4. In the multivariable analysis, the only variable that emerged with significance was the daily number of milking sessions (*p* = 0.049) (Figure 3, Table 4).

#### 3.3.4. Teatcups with Tears or Cracks

We have presented in Table S5 all of the details of the univariable analysis for associations of the variables assessed with the presence of tears or cracks on the teatcups. In the multivariable analysis, the following variable emerged with a significance value: the vacuum level of the milking system (*p* = 0.017) (Table 5).

## 4. Discussion

### 4.1. Frequency of Irregular Findings on Teatcups and Predictors

The results have indicated that irregular findings were recorded in almost half of the farms in which the teatcups were macroscopically assessed. The higher proportion of teatcups with dirt or milk residues (in comparison to worn teatcups) indicates that cleaning procedures in the respective milking parlours were erratically performed. Hence, the post-milking management at the milking parlour of those farms must be improved.

The results of the analysis for predictors suggest that overuse of the milking system is responsible for the presence of irregular findings on the teatcups. This issue can occur through various means: the high number (three) of milking sessions performed daily, the advanced stage (beyond the 5th month) of the lactation period and the multiple use of milking units.

For the first variable (i.e., the daily number of milking sessions), it becomes evident that due to smaller intervals between milking sessions when frequent milking sessions daily take place, the cleaning procedures may possibly be hastily performed. This issue can lead to accumulation of milk residues on the teatcups, as was more clearly found in the analysis of this specific finding.

For the second variable (i.e., the month into the lactation period at sampling), it is suggested that the accumulation of dirt (which likely originates from the teats of the animals) on the teatcups progressively takes place as the lactation period advances and as the milking system is used again and again with suboptimal post-milking cleaning. Once again, this issue was more clearly shown in the analysis of this specific finding.

For the third variable (i.e., the number of available milking units per animal position), we may postulate that the units within a milking system are often disproportionately used for the number of animals in the farm (which fit into the positions within a parlour, but no milking units would be correspondingly available to each position). Moreover, in such cases, a longer period of time is also necessary for a milking session, as units need to be transferred from the animal in one position to the animal in the next one.

The season when visits to the farms were carried out was found, in the univariable analysis, to be significant, specifically noting a higher frequency of teatcups with irregular findings during the summer. This issue can be considered to be the consequence of longer grazing periods of animals during the summer and, consequently, more chances for the teats to become dirty. This finding allies well with the importance of the month into the lactation period, as discussed above; in Greece, the reproductive season primarily takes place during the summer [11]; therefore, lambings/kiddings in most farms would start in the autumn or early winter, and, thus, at the beginning of the summer, animals would be in the 7th to 9th month into the lactation period.

It is noted that in cattle, additional factors have been identified that influence the condition of teatcups post-milking, e.g., the temperature of the water used for the washing of the milking system, use of detergent, schedule for the maintenance of the milking system and the replacement of teatcups, etc. Several of these factors have been assessed in the present study but were not found to be significant. There also significant differences between cattle and sheep farms as, for example, in cows, careful cleaning and washing of teats is performed, which significantly reduces dirt and bacterial burden, or the immersion of the teatcups into water and detergent occurs after the end of the milking procedure [12]; these practices are not performed in small ruminant farms.

Poor cleaning management of the milking parlour will contribute to increased bacterial burdens in the animal environment and, consequently, increased risk of infection and, finally, potential for the development of mastitis [13]. This issue will, in turn, lead to increased cell content in milk, which is characterised by inflammation, as well as bacterial shedding in milk. Finally, these issue will result in high somatic cell counts and total bacterial counts in the bulk-tank milk of the farm.

### 4.2. Association with Increased Isolation of Staphylococci from Teatcups

The removal of milk residues from teatcups has already been associated with reduced bacterial populations on teatcups [14] and, therefore, the accumulation of dirt or milk residues on the teatcups provides a suitable substrate for bacteria to grow and multiply. The bacteria on teatcups can be transferred to the teats of ewes/female goats during the subsequent milking, which increases the risk of intramammary infections.

Nevertheless, the less frequent isolation of staphylococci from teatcups with tears or cracks was not expected. Cracks and fissures present on used materials [15] lead to increases in their total surface (compared to brand-new teatcups, which have an intact and smooth surface). These fissures may contain bacteria, thus potentially increasing the total bacterial loads on such materials, given that the amount of staphylococci adhering to coarse material is higher than that on fine surfaces. The current results ally well with previous findings recently presented, in which it was shown that the speed of the dissemination of staphylococci on teatcups did not significantly differ between brand-new and used ones [16].

The present study focused on associations of irregular findings on teatcups with the isolation of staphylococci from these tools, as these bacteria are the primary pathogens of interest in ovine and caprine mastitis. Nevertheless, other bacteria may be isolated from cases of the infection, even at a lower frequency (e.g., streptococci). In a previous smaller study, we entirely focused on the recovery of streptococci from teatcups from milking parlours in small ruminant farms. Streptococci (mainly *Streptococcus uberis*) were recovered from only 6.0% of 251 teatcups from the milking parlours of 7 of the 55 farms studied [17]. In order to compare them to the findings of the present work, a meta-analysis of a possible association between the recovery of streptococci from the teatcups and the presence of irregular findings on the teatcups was performed, which did not reveal a significant association between the two variables (*p* = 0.73), in sharp contrast to the results of the present work, which have shown a clear association with the isolation of staphylococci. In sheep and goats, streptococci are infrequent causal agents of mastitis, and the infrequent isolation of these bacteria from teatcups is in line with this fact.

## 5. Conclusions

The study provides, for the first time internationally, an appraisal of the frequency of problematic teatcups in the milking parlours of small ruminant farms. The analysis of predictors has provided a focus for specific management aspects, where interventions need to be performed, in order to improve the situation in farms with problems. The consequent increased staphylococcal burden on teatcups with irregular findings points to the increased risk of intramammary infections in such cases.

## Figures and Tables

**Table 1:** Types of macroscopically evident irregular findings observed on teatcups in milking parlours in 255 sheep flocks and 66 goat herds in a country-wide investigation in Greece.

Type of Irregular Findings	Sheep Flocks (*n* = 119)	Goat Herds (*n* = 31)	*p*-Value
Presence of dirt	106 (89.1%)	29 (93.5%)	0.46
Presence of milk residues	44 (37.0%)	10 (32.3%)	0.63
Presence of cracks or tears	14 (11.8%)	5 (16.1%)	0.52

**Table 2:** Frequency of isolation of staphylococcal species from milking parlours in sheep and goat farms in a country-wide investigation in Greece, in accordance with the type of irregular findings observed on the teatcups.

	Type of Irregular Findings on Teatcups			
Staphylococcal Species	Presence of Dirt	Presence of Milk Residues	Presence of Cracks or Tears	*p*-Value
*S. aureus*	22 (25.6%)	9 (25.7%)	3 (30.0%)	0.95
*S. auricularis*	1 (1.2%)	1 (2.9%)	0 (0.0%)	0.73
*S. capitis*	3 (3.5%)	1 (2.9%)	1 (10.0%)	0.56
*S. chromogenes*	3 (3.5%)	1 (2.9%)	0 (0.0%)	0.83
*S. cohnii* subsp. *cohnii*	2 (2.3%)	0 (0.0%)	1 (10.0%)	0.18
*S. cohnii* subsp. *urealyticum*	2 (2.3%)	0 (0.0%)	0 (0.0%)	0.59
*S. epidermidis*	2 (2.3%)	1 (2.9%)	0 (0.0%)	0.87
*S. equorum*	3 (3.5%)	2 (5.7%)	0 (0.0%)	0.68
*S. haemolyticus*	8 (9.3%)	4 (11.4%)	0 (0.0%)	0.54
*S. intermedius*	1 (1.2%)	1 (2.9%)	0 (0.0%)	0.73
*S. kloosii*	5 (5.8%)	3 (8.6%)	1 (10.0%)	0.79
*S. lentus*	5 (5.8%)	0 (0.0%)	0 (0.0%)	0.26
*S. lugdunensis*	5 (5.8%)	2 (5.7%)	2 (20.0%)	0.23
*S. pettenkoferi*	1 (1.2%)	0 (0.0%)	0 (0.0%)	0.77
*S. saprophyticus*	1 (1.2%)	0 (0.0%)	0 (0.0%)	0.77
*S. sciuri*	2 (2.3%)	2 (5.7%)	0 (0.0%)	0.52
*S. simulans*	15 (17.4%)	3 (8.6%)	1 (10.0%)	0.42
*S. vitulinus*	2 (2.3%)	2 (5.7%)	0 (0.0%)	0.52
*S. warneri*	1 (1.2%)	2 (5.7%)	1 (10.0%)	0.17
*S. xylosus*	2 (2.3%)	1 (2.9%)	0 (0.0%)	0.87
Total	86	35	10	

**Table 3:** Results of multivariable analysis of associations of the presence of teatcups with macroscopic irregular findings in milking parlours in 255 sheep flocks and 66 goat herds in Greece.

Variables	Odds Risk (±se)/ Odds Ratios (95% CI) ^1^	*p*
Daily number of milking sessions		0.033
One (1/3 = 33.3%)	reference	-
Two (144/246 = 43.8%)	2.571 (0.230–28.721)	0.44
Three (36/61 = 59.0%)	2.880 (0.248–33.513)	0.40
Month into the lactation period at sampling		0.046
Per unit increase	1.020 ± 1.010	-
Number of available milking units per animal position		0.047
Less than one	1.474 (0.916–2.374)	0.11
One	reference	-

^1^: se: standard error, CI: confidence interval.

**Table 4:** Results of multivariable analysis for associations with the presence of teatcups with milk residues in milking parlours in 255 sheep flocks and 66 goat herds in Greece.

Variables	Odds Ratios (95% CI) ^1^	*p*
Daily number of milking sessions		0.049
One (0/3 = 0.0%)	reference	-
Two (39/257 = 15.2%)	1.265 (0.064–24.978)	0.88
Three (15/61 = 59.7%)	2.333 (0.114–47.741)	0.58

^1^: CI: confidence interval.

**Table 5:** Results of multivariable analysis for associations of the presence of teatcups with tears or cracks in milking parlours in 255 sheep flocks and 66 goat herds in Greece.

Variable	Odds Ratios	*p*
Vacuum level of the milking system		0.017
<38 kPa (2/27 = 7.4%)	1.984 (0.412–9.564)	0.39
38–42 kPa (10/258 = 3.9%)	reference	-
>42 kPa (7/36 = 19.4%)	5.986 (2.117–16.931)	0.0007

## Data Availability

Most data presented in this study are presented in the Supplementary Materials. The remaining data are available on request from the corresponding author. The data are not publicly available as they form part of the PhD thesis of the first author, which has not yet been examined, approved and uploaded in the official depository of PhD theses from Greek Universities.

## Supplementary Materials

The following supporting information can be downloaded via this link: https://www.mdpi.com/article/10.3390/ani13193004/s1, Table S1: Details of multivariable models employed for the evaluation of predictors of the recovery of staphylococcal isolates from teatcups in milking parlours in 255 sheep flocks and 66 goat herds in Greece; Table S2. Results (frequencies) of univariable analysis of variables evaluated for association with the outcome ‘presence of teatcups with macroscopic abnormal findings’ in the milking parlours of 255 sheep flocks and 66 goat herds in Greece; Table S3. Results (frequencies) of univariable analysis of variables evaluated for association with the outcome ‘presence of teatcups with dirt’ in the milking parlours of 255 sheep flocks and 66 goat herds in Greece; Table S4. Results (frequencies) of univariable analysis of variables evaluated for association with the outcome ‘presence of teatcups with milk residues’ in the milking parlours of 255 sheep flocks and 66 goat herds in Greece; Table S5. Results (frequencies) of univariable analysis of variables evaluated for association with the outcome ‘presence of teatcups with cracks or tears’ in the milking parlours of 255 sheep flocks and 66 goat herds in Greece.

## Appendix A. Variables Evaluated for Potential Association with the Presence of Irregular Findings on Teatcups in Milking Parlours in 255 Sheep Flocks and 66 Goat Herds in Greece

**Parameters Related to Sampling Conditions**
Season of the year in which sampling was performed (season)
Month into the lactation period at sampling (month)
**Parameters Related to the Management and the Health and Production in the Flock/Herd**
Management system applied to the flocks/herds (description according to EFSA classification)
No. of ewes/does in the flocks/herds (no.)
Average age of culling females (years)
Daily number of milking sessions (no.)
Application of post-milking teat disinfection (yes/no)
Annual milk production per animal (L)
**Parameters Related to the Milking Parlour in the Flock/Herd**
Years since initial establishment or most recent renovation of the milking parlour (no.)
Volume of the parlour (m^3^)
Material of the floor of the milking parlour (cement/tile/soil/other)
Type of milking parlour (fishbone/circular/linear parallel/linear one-sided/other)
Type of milking system (mobile/non-mobile)
Number of animal positions in the parlour (no.)
Number of available milking units per animal position (no.)
Provision of feed in the parlour (yes/no)
Availability of automated milk quantity measurement (yes/no)
Availability of milk quality indicators (yes/no)
Availability of milk flow indicators (yes/no)
System pulsation rate (p. min^−1^)
System vacuum level (kPa.)
System pulsation rate-to-vacuum level ratio
Type of flow line (low/high/other)
Frequency of check-ups of the system by farmer (description)
Annual frequency of check-ups of the system by technicians (no. of occasions)
Water cleaning of parlour after each milking session (yes/no)
Temperature of cleaning water (°C)
Use of detergent for parlour cleaning after the milking sessions (yes/no)
Frequency of changing teatcups (description)
**Parameters Related to the Socio-demographic Characteristics of Farmers**
Age of the farmer (years)
Length of animal farming experience of the farmer (years)
Education of the farmer (description)
Presence of working staff in the flock (yes/no)

## Appendix B. Statistical Significance Values for Variables Assessed via the Univariable Analysis of the Presence of Irregular Findings on Teatcups in Milking Parlours in 255 Sheep Flocks and 66 Goat Herds in Greece

**Variables**	***p*-Value**
**Parameters Related to Sampling Conditions**	
Season of the year when sampling was performed	0.0009
Month into the lactation period at sampling	0.017
**Parameters Related to the Management and the Health and Production in the Flock/Herd**	
Management system applied to the flocks	0.27
No. of ewes/does in the flocks/herd	0.16
Average age of culling females	0.82
Daily number of milking sessions	0.10
Application of post-milking teat disinfection	0.26
Annual milk production per animal	0.45
**Parameters Related to the Milking Parlour in the Flock/Herd**	
Years since initial establishment or most recent renovation of the milking parlour	0.40
Volume of the parlour	0.93
Material of the floor of the milking parlour	0.79
Type of milking parlour	0.67
Type of milking system	0.90
Number of animal positions in the parlour	0.66
Number of available milking units per animal position	0.11
Provision of feed in the parlour	0.83
Availability of automated milk quantity measurement	0.84
Availability of milk quality indicators	n/a
Availability of milk flow indicators	0.25
System pulsation rate	0.16
System vacuum level	0.45
System pulsation rate-to-vacuum level ratio	0.73
Type of flow line	0.21
Frequency of check-ups of the system by farmer	0.21
Annual frequency of check-ups of the system by technicians	0.33
Water cleaning of parlour after each milking session	0.14
Temperature of cleaning water	0.44
Use of detergent for parlour cleaning after the milking sessions	0.64
Frequency of changing teatcups	0.20
**Parameters Related to the Socio-demographic Characteristics of Farmers**	
Age of the farmer	0.34
Length of previous animal farming experience	0.034
Education of the farmer	0.34
Presence of working staff in the flock	0.93
