# Supplementary material for: Irregular Findings on Teatcups in Milking Parlours in Sheep and Goat Farms and Potential Predictors
Source: Animals (Basel). 2023 Sep 23;13(19):3004. doi: 10.3390/ani13193004 (PMC10571646; doi:10.3390/ani13193004)
Supplement: Supplementary file 1 [file animals-13-03004-s001.zip › animals-2605444-supplementary.pdf]

Irregular Findings on Teatcups of Milking Parlours in Sheep and Goat Farms and Potential Predictors

Charalambia K. Michael, Daphne T. Lianou, Vasia S. Mavrogianni, Efthymia Petinaki and George C. Fthenakis

Table S1. Details of multivariable models employed for the evaluation of predictors for the presence of irregular findings on teatcups of milking parlours in 255 sheep flocks and 66 goat herds in Greece.

Outcome	Variables offered to the multivariable models (*n*)	Variables required in the final models
Presence of teatcups with irregular findings	8	(a) Month into the lactation period at sampling, (b) No. of ewes / does in the flocks / herds, (c) Daily number of milking sessions, (d) Number of available milking units per animal position, (e) System pulsation rate, (f) Water cleaning of parlour after the milking sessions, (g) Length of animal farming experience of the farmer
Presence of teatcups with dirt	7	(a) Month into the lactation period at sampling, (b) No. of ewes / does in the flocks / herds, (c) Number of available milking units per animal position, (d) Water cleaning of parlour after the milking sessions, (e) Length of animal farming experience of the farmer
Presence of teatcups with milk residues	2	(a) Season of the year when sampling was performed (b) Daily number of milking sessions
Presence of teatcups with cracks or tears	2	(a) Month into the lactation period at sampling, (b) System vacuum level

Table S2. Results (frequencies) of univariable analysis of variables evaluated for association with the outcome 'presence of teatcups with irregular findings' in the milking parlours of 255 sheep flocks and 66 goat herds in Greece.

Farms in which no teatcups with irregular findings were found (*n* = 171)				Farms in which teatcups with irregular findings were found (*n* = 150)				
**Season of the year when sampling was performed**								
Spring	Summer	Autumn	Winter	Spring	Summer	Autumn	Winter	*p*
60	43	21	47	28	66	16	40	0.0009
**Month into the lactation period at sampling**								
5.0 ± 0.2 months				5.8 ± 0.2 months				0.017
**Management system applied in farms**								
Intensive	Semi-intensive	Semi-extensive	Extensive	Intensive	Semi-intensive	Semi-extensive	Extensive	*p*
28	73	68	2	24	76	46	4	0.27
**No. of ewes / does in the flock / herd**								
308 ± 17 animals				347 ± 23 animals				0.16
**Average age of culling females**								
5.9 ± 0.1 years				5.9 ± 0.1 years				0.82
**Daily number of milking sessions**								
One	Two	Three		One	Two	Three		*p*
2	144	25		1	113	36		0.10
**Application of post-milking teat disinfection**								
Yes	No			Yes	No			*p*
37	134			25	125			0.26
**Annual milk production per animal**								
231 ± 7 L				223 ± 7 L				0.45
**Years since initial establishment or most recent renovation of the milking parlour**								
7.5 ± 0.5 years				8.1 ± 0.5 years				0.40

**Volume of the parlour**								
201 ± 12 m^3				199 ± 12 m^3				0.93
**Material of the floor of the milking parlour**								
Concrete	Tiles	Soil	Other	Concrete	Tiles	Soil	Other	*p*
94	27	29	21	82	29	21	18	0.79
**Type of milking parlour**								
Fishbone	Circular	Linear parallel	Linear one-sided	Fishbone	Circular	Linear parallel	Linear one-sided	*p*
1	6	61	103	0	8	51	91	0.67
**Type of milking system**								
Mobile		Built-in		Mobile		Built-in		*p*
22		149		20		130		0.90
**Number of animal positions in the parlour**								
Less than 24	24	Over 24		Less than 24	24	Over 24		*p*
63	70	38		48	67	35		0.66
**Number of available milking units per animal position**								
< 1		1		< 1		1		*p*
110		61		109		41		0.11
**Provision of feed in the parlour**								
Yes		No		Yes		No		*p*
154		17		134		16		0.83
**Availability of automated milk quantity measurement**								
Yes		No		Yes		No		*p*
10		161		8		142		0.84

**Availability of milk quality indicators**						
Yes	No		Yes	No		*p*
0	171		0	150		n/a
**Availability of milk flow indicators**						
Yes	No		Yes	No		*p*
1	170		3	147		0.25
**System pulsation rate**						
< 140 p. min^{-1}	140 - 150 p. min^{-1}	> 150 p. min^{-1}	< 140 p. min^{-1}	140 - 150 p. min^{-1}	> 150 p. min^{-1}	*p*
36	98	37	42	86	22	0.16
**System vacuum level**						
< 38 kPa	38 - 42 kPa	> 42 kPa	< 38 kPa	38 - 42 kPa	> 42 kPa	*p*
16	133	22	11	125	14	0.45
**System pulsation rate to vacuum level ratio**						
	(3.7 ± 0.1) : 1			(3.7 ± 0.1) : 1		0.73
**Type of flow line**						
High	Low	Other	High	Low	Other	*p*
127	32	12	98	39	13	0.21
**Frequency of check-ups of the system by farmer**						
Daily	Less frequently than daily		Daily	Less frequently than daily		*p*
142	29		132	18		0.21
**Annual frequency of check-ups of the system by technicians**						
< 1 check-up	1 - 2 check-ups	> 2 check-ups	< 1 check-up	1 - 2 check-ups	> 2 check-ups	*p*
31	116	24	21	100	29	0.33
**Water cleaning of parlour after the milking sessions**						
Yes	No		Yes	No		*p*
164	7		148	2		0.14

Temperature of cleaning water				
< 50 °C	≥ 50 °C	< 50 °C	≥ 50 °C	*p*
8	163	10	140	0.44
**Use of detergent for parlour cleaning after the milking sessions**				
Yes	No	Ye	No	*p*
169	2	149	1	0.64
**Frequency of changing teatcups**				
Up to annually	Less frequently than annually	Up to annually	Less frequently than annually	*p*
79	92	80	70	> 0.20
**Age of the farmer**				
45.0 ± 0.9 years		46.2 ± 0.9 years		0.34
**Length of previous animal farming experience**				
20.2 ± 1.2 years		24.0 ± 1.3 years		0.034

Education of farmer						
Primary	Secondary and post-secondary	Tertiary	Primary	Secondary and post-secondary	Tertiary	*p*
27	118	26	19	114	17	0.34

Presence of working staff in the farm				
Yes	No	Yes	No	*p*
76	95	66	84	0.93

Table S3. Results (frequencies) of univariable analysis of variables evaluated for association with the outcome 'presence of teatcups with dirt' in the milking parlours of 255 sheep flocks and 66 goat herds in Greece.

**Farms in which no teatcups with dirt were found (*n* = 186)**				**Farms in which teatcups with dirt were found (*n* = 135)**				
**Season of the year when sampling was performed**								
Spring	Summer	Autumn	Winter	Spring	Summer	Autumn	Winter	*p*
63	48	26	49	25	61	11	38	<0.0001
**Month into the lactation period at sampling**								
5.5 ± 0.2 months				6.0 ± 0.2 months				0.002
**No. of ewes/does in the flock / herd**								
309 ± 13 animals				350 ± 25 animals				0.15
**Daily number of milking sessions**								
One	Two	Three		One	Two	Three		*p*
2	154	30		1	103	31		0.30
**Number of available milking units per animal position**								
< 1	1			< 1	1			*p*
121	65			98	37			0.15
**System pulsation rate**								
< 140 p. min^{-1}	140 - 150 p. min^{-1}	> 150 p. min^{-1}		< 140 p. min^{-1}	140 - 150 p. min^{-1}	> 150 p. min^{-1}		*p*
40	108	38		38	76	21		0.29
**System vacuum level**								
< 38 kPa	38 - 42 kPa	> 42 kPa		< 38 kPa	38 - 42 kPa	> 42 kPa		*p*
17	143	26		10	115	10		0.14
**Water cleaning of parlour after the milking sessions**								
Yes	No			Yes	No			*p*
178	8			134	1			0.06

**Length of previous animal farming experience**		
20.7 ± 1.0 years	23.5 ± 1.4 years	0.11

Table S4. Results (frequencies) of univariable analysis of variables evaluated for association with the outcome 'presence of teatcups with milk residues' in the milking parlours of 255 sheep flocks and 66 goat herds in Greece.

Farms in which no teatcups with milk residues were found (n = 267)				Farms in which teatcups with milk residues were found (n = 54)				
**Season of the year when sampling was performed**								
Spring	Summer	Autumn	Winter	Spring	Summer	Autumn	Winter	*p*
79	84	32	72	9	25	5	15	0.11
**Month into the lactation period at sampling**								
5.4 ± 0.2 months				5.4 ± 0.4 months				0.94
**No. of ewes / does in the flock / herd**								
327 ± 16 animals				322 ± 27 animals				0.90
**Daily number of milking sessions**								
One	Two	Three		One	Two	Three		*p*
3	218	46		0	39	15		0.15
**Number of available milking units per animal position**								
< 1	1			< 1	1			*p*
183	84			36	18			0.79
**System pulsation rate**								
< 140 p. min^{-1}	140 - 150 p. min^{-1}	> 150 p. min^{-1}		< 140 p. min^{-1}	140 - 150 p. min^{-1}	> 150 p. min^{-1}		*p*
62	156	49		16	28	10		0.58
**System vacuum level**								
< 38 kPa	38 - 42 kPa	> 42 kPa		< 38 kPa	38 - 42 kPa	> 42 kPa		*p*
23	213	31		4	45	5		0.83
**Water cleaning of parlour after the milking sessions**								
Yes	No			Yes	No			*p*
259	8			53	1			0.64

**Length of previous animal farming experience**		
21.6 ± 0.9 years	23.7 ± 2.3 years	0.37

Table S5. Results (frequencies) of univariable analysis of variables evaluated for association with the outcome 'presence of teatcups with cracks or tears' in the milking parlours of 255 sheep flocks and 66 goat herds in Greece.

**Farms in which no teatcups with cracks or tears were found (*n* = 302)**				**Farms in which teatcups with cracks or tears were found (*n* = 19)**				
**Season of the year when sampling was performed**								
Spring	Summer	Autumn	Winter	Spring	Summer	Autumn	Winter	*p*
86	102	33	81	2	7	4	6	0.27
**Month into the lactation period at sampling**								
5.5 ± 0.2 months				4.3 ± 0.9 months				0.07
**No. of ewes / does in the flock / herd**								
326 ± 14 animals				326 ± 76 animals				0.99
**Daily number of milking sessions**								
One	Two	Three		One	Two	Three		*p*
3	243	56		0	14	5		0.65
**Number of available milking units per animal position**								
< 1	1			< 1	1			*p*
207	95			12	7			0.62
**System pulsation rate**								
< 140 p. min^{-1}	140 - 150 p. min^{-1}	> 150 p. min^{-1}		< 140 p. min^{-1}	140 - 150 p. min^{-1}	> 150 p. min^{-1}		*p*
74	171	57		4	13	2		0.55
**System vacuum level**								
< 38 kPa	38 - 42 kPa	> 42 kPa		< 38 kPa	38 - 42 kPa	> 42 kPa		*p*
25	248	29		2	10	7		0.001
**Water cleaning of parlour after the milking sessions**								
Yes	No			Yes	No			*p*
293	9			19	0			0.45

**Length of previous animal farming experience**		
21.9 ± 0.9 years	22.3 ± 5.3 years	0.93
